# Impact of hippocampal α‐synuclein oligomers on cognitive trajectory in patients with dementia with Lewy bodies

**DOI:** 10.1002/alz.70374

**Published:** 2025-08-04

**Authors:** Hiroaki Sekiya, Lukas Franke, Daisuke Ono, Michael DeTure, Owen A. Ross, Melissa E. Murray, Pamela J. McLean, Tanis J. Ferman, Dennis W. Dickson

**Affiliations:** ^1^ Department of Neuroscience Mayo Clinic Jacksonville Florida USA; ^2^ Department of Laboratory Medicine and Pathology Mayo Clinic Jacksonville Florida USA; ^3^ Department of Psychiatry & Psychology Mayo Clinic Jacksonville Florida USA

**Keywords:** dementia with Lewy bodies, oligomers, α‐synuclein

## Abstract

**INTRODUCTION:**

Increasing evidence indicates that α‐synuclein (αSYN) oligomers are toxic. We sought to determine whether αSYN oligomers were associated with faster cognitive decline in prospectively‐followed patients with dementia with Lewy bodies (DLB).

**METHODS:**

Eight autopsy‐confirmed patients with DLB were selected based on rapid or slow cognitive decline determined by the rate of change of Mini‐Mental State Examination (MMSE) scores. Quantitative neuropathologic analysis of αSYN oligomers, Lewy‐related pathology, phosphorylated tau, and amyloid‐β was conducted in hippocampal subfields (CA1‐4 and subiculum) and the entorhinal cortex.

**RESULTS:**

DLB with rapid cognitive decline showed greater CA1 αSYN oligomer burden (*p* = 0.029) and tau burden (*p* = 0.029) than DLB with slow decline. The groups showed comparable burden of Lewy‐related pathology and amyloid‐β pathology in the hippocampal formation and entorhinal cortex.

**DISCUSSION:**

Hippocampal accumulation of αSYN oligomers and phosphorylated tau is associated with rapid cognitive decline in DLB. Therapeutic strategies targeting αSYN oligomers warrant further investigation.

**Highlights:**

- Proximity ligation assay (PLA) and digital pathology for oligomer quantification.
- Abundant α‐synuclein (αSYN) oligomers in CA1 of patients with dementia with Lewy bodies (DLB) with rapid decline.
- First human brain study linking αSYN oligomers to cognitive trajectory.
- Potential therapeutic implications of targeting αSYN oligomers in DLB.

## 1 INTRODUCTION

Dementia with Lewy bodies (DLB) is the second most common neurodegenerative dementia after Alzheimer's disease (AD) dementia. Clinical features include dementia with parkinsonism, visual hallucinations, cognitive fluctuations, autonomic dysfunction, and sleep disorders.^1^ The neuropathologic hallmark of DLB is the presence of aggregated α‐synuclein (αSYN) in neurons and in dystrophic neurites, together referred to as Lewy‐related pathology.^2^, ^3^, ^4^ During the aggregation of αSYN, oligomeric forms appear prior to the formation of mature Lewy‐related pathology.^5^ These early aggregates are considered more toxic than Lewy‐related pathology and may be involved in the pathogenesis of α‐synucleinopathies.^6^, ^7^, ^8^ Although detection of αSYN oligomers in pathology specimens can be difficult, the application of a proximity ligation assay (PLA) has enabled their visualization.^9^, ^10^ Previous studies have shown associations between αSYN oligomers and cognitive impairment in various α‐synucleinopathies.^11^, ^12^ However, the relationship between the progression rate of cognitive decline and αSYN oligomer accumulation remains unclear.

The present study aimed to investigate the relationship between the rate of cognitive decline and the accumulation of αSYN aggregates, including both oligomeric and fibrillar forms, in hippocampal subfields of DLB patients who had undergone longitudinal cognitive assessments. We hypothesized that the distribution and quantity of αSYN oligomers, rather than mature Lewy‐related pathology, would be associated with the rate of cognitive deterioration.

## 2 MATERIALS AND METHODS

### 2.1 Subjects

**FIGURE 1:**
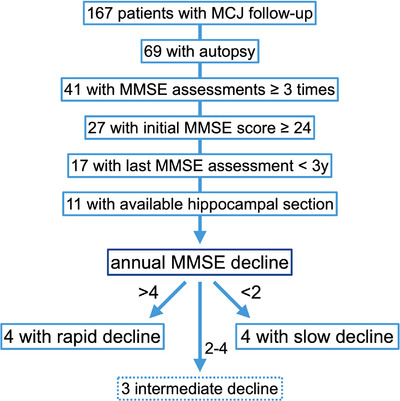
Study cohort. MCJ, Mayo Clinic Jacksonville; MMSE, Mini‐Mental Status Examination.

Figure 1A schematically illustrates case selection. In order to compare the underlying neuropathology between patients with rapid and slow cognitive decline, the following inclusion criteria were applied: (1) at least three Mini‐Mental State Examination (MMSE) evaluations, (2) MMSE score at the initial visit of at least 24 points, (3) the last MMSE examination within 3 years of death, and (4) availability of a tissue sample from the posterior hippocampus.

### 2.2 Assessment of cognitive decline

The rate of change in the MMSE score per unit of time between examinations was calculated using the slope function as previously described.^13^ This value represents an annualized rate of change from the initial MMSE to the last MMSE.

### 2.3 Neuropathologic evaluation

All cases underwent a standardized neuropathologic evaluation by a neuropathologist (D.W.D.) that included both macroscopic and microscopic assessments. The microscopic evaluation included hematoxylin and eosin staining, thioflavin S fluorescent microscopy, and immunohistochemistry with an antibody against αSYN (NACP, rabbit polyclonal, 1:3000, with formic acid pretreatment) as previously described.^14^, ^15^ Braak neurofibrillary tangle stage and Thal amyloid phase were assigned based upon thioflavin S fluorescent microscopy. A neuropathologic diagnosis of Lewy body disease was established based on the identification of Lewy bodies in vulnerable brain regions.^3^ Lewy body disease subtypes were further categorized as amygdala‐predominant, brainstem, transitional (limbic), and diffuse (neocortical) types based on the severity and distribution of Lewy‐related pathology.^1^, ^16^

Comorbid pathologies including transactive response DNA binding protein of 43 kDa (TDP‐43) pathology and cerebral small vessel disease were assessed as previously described.^17^ TDP‐43 pathology was assessed with immunohistochemistry with phosphorylated TDP‐43 antibody (pS409/410, mouse monoclonal, 1:5000, CosmoBio USA, Carlsbad, CA). The section at the level of the anterior commissure, including the amygdala, basal forebrain, putamen, globus pallidus, and hypothalamus was used for screening of TDP‐43 pathology. Cerebral small vessel disease was assessed on hematoxylin and eosin–stained sections and considered positive if there were arteriolosclerosis with microinfarcts, microbleeds, or ischemic white matter changes.

### 2.4 Brain regions of interest

To investigate the underlying neuropathologic substrates of cognitive decline, we examined the hippocampus and entorhinal cortex for αSYN pathology (both early‐ and late‐stage aggregates), amyloid‐β, and tau pathology. Within the hippocampus, we analyzed subfields including CA1, CA2, CA3, CA4, and subiculum.

Neuronal loss was assessed semi‐quantitatively on hematoxylin and eosin–stained sections in each region using a 4‐point scale (0: none, 1: mild, 2: moderate, and 3: severe). Two researchers (H.S. and D.W.D.), blinded to clinical information, performed the assessment using a multi‐head microscope.

### 2.5 Immunohistochemistry

Immunohistochemistry was performed on an autostainer (Thermo Lab Vision Autostainer 480S), using EnVision+ reagents (Dako, Carpinteria, CA, USA). For Lewy‐related pathology, phosphorylated‐αSYN antibody (1:10,000; mouse monoclonal, psyn#64, FUJIFILM Wako Pure Chemical Corporation, Osaka, Japan) was used. For amyloid β pathology, sections were stained with 4G8 antibody including 95% formic acid pretreatment (1:50000, mouse monoclonal, BioSource International/Invitrogen Corp., Carlsbad, CA, USA). For phosphorylated tau pathology, sections were stained with PHF‐1 antibody (1:1000, mouse monoclonal, gift from the late Dr. Peter Davies; Feinstein Institute for Medical Research).^18^

### 2.6 αSYN‐PLA staining

To visualize αSYN oligomers, αSYN‐PLA staining was used as previously described, with minor modifications.^19^, ^20^ The procedure was performed on 5‐µm‐thick sections using Duolink kits (Sigma‐Aldrich, St. Louis, MO, USA) in accordance with the manufacturer's instructions. For PLA probe preparation, we used an αSYN antibody (Syn211, mouse monoclonal, Abcam, Cambridge, UK). Specifically, we added 20 µg of Syn211 antibody to 2 µL of conjugation buffer and transferred the solution to a vial containing lyophilized oligonucleotides (plus or minus). Subsequently, the mixture was left to incubate overnight at room temperature. The conjugates were then incubated with 2 µL of stop solution for 30 min at room temperature and suspended in 24 µL of storage solution. Following the dewaxing and hydrating of tissue sections as described above, the sections were steamed in Target Retrieval Solution (pH 6, Agilent, Santa Clara, CA, USA) for 30 min, followed by blocking in Duolink blocking solution at 37°C for 1 h. This was followed by incubation with PLA probes diluted in PLA probe diluent (1:400) at 37°C for 1 h and then at 4°C overnight. After washing the sections in wash buffer A, they were incubated with ligation solution and ligase at 37°C for 1 h, followed by washing in wash buffer A and incubation with amplification reagents and polymerase at 37°C for 2.5 h. Finally, the sections were washed in wash buffer A, incubated with detection solution at room temperature for 1 h, and subsequently incubated with substrate solution at room temperature for 20 min. The sections were counter‐stained with hematoxylin, dehydrated in a graded series of alcohol and xylene, and then mounted with a bright‐field mounting medium.

**RESEARCH IN CONTEXT**

1. **Systematic review**: We conducted a PubMed search using the terms “DLB” and “synuclein oligomer”. While several studies have investigated α‐synuclein (αSYN) oligomers in rodent models or human cerebrospinal fluid, we found no studies that evaluated the relationship between αSYN oligomers and the rate of cognitive decline using autopsy brain tissue from patients with dementia with Lewy bodies (DLB).
2. **Interpretation**: Our findings demonstrate that patients with DLB who show rapid cognitive decline have more abundant αSYN oligomers in the hippocampal CA1 region compared to those with slow decline. These results provide the first direct evidence from human brain tissue that αSYN oligomers may influence the cognitive decline trajectory in DLB.
3. **Future directions**: Our findings warrant further investigation in larger cohorts to determine whether αSYN oligomers and tau pathology have independent or synergistic effects on cognitive decline. Our results suggest that αSYN oligomers could be therapeutic targets for modifying cognitive decline in DLB.

The validity of αSYN‐PLA staining for detecting αSYN oligomers has been established through multiple approaches. These include confirmation of αSYN oligomers visualized via bimolecular fluorescence complementation and rapamycin‐induced αSYN dimerization in an FKBP‐FRB system, and selective recognition of oligomeric αSYN over monomeric and fibrillar forms in synthesized αSYN preparations.^9^ Additionally, Western blot analysis has confirmed the presence of higher molecular weight αSYN species corresponding to those detected by αSYN‐PLA staining.^9^, ^10^

### 2.7 Quantitative neuropathologic analysis

Quantitative neuropathologic analysis of αSYN oligomers, Lewy‐related pathology, amyloid β, and phosphorylated tau was performed in the hippocampus and entorhinal cortex as previously described.^18^ Stained slides were digitally scanned at x20 magnification using the Aperio ScanScope AT2 (Leica Biosystems, Buffalo Grove, IL, USA). Scanned images were viewed and annotated using ImageScope (version 12.4.3; Leica Biosystems, Deer Park, IL, USA) and QuPath^21^ (version 0.5.0). A region of interest was outlined for each brain region. Custom algorithms were used for quantitative analysis: a positive pixel count algorithm for 4G8 and a color deconvolution algorithm for phosphorylated αSYN and PHF‐1 immunostaining to quantify strongly 3,3'‐diaminobenzidine (DAB)‐immunoreactive pixels. For αSYN‐PLA staining, strongly stained NovaRed‐positive pixels were quantified using a custom positive pixel count algorithm. This analysis yielded a percentage burden reflecting the proportion of immunoreactive pathology within the total area examined. All quantitative neuropathologic analyses were performed by investigators blinded to the clinical status of the cases.

### 2.8 Genetic information

We extracted genomic DNA from the frozen cerebellar tissue using the AutoGen 245 T platform. Apolipoprotein E (*APOE)* genotype was determined based on the two single nucleotide polymorphisms, rs7412 and rs429358.

### 2.9 Statistics

Statistical analyses were performed with GraphPad Prism (version 10.1.0, GraphPad Software, La Jolla, CA, USA). Fisher's exact test was used to compare categorical variables. Due to the small sample size, the Mann–Whitney test was conducted for all continuous variables, including the comparison of stained areas between rapid and slow cognitive decline groups. Spearman's rank correlation coefficient was calculated between the stained areas of αSYN oligomers and phosphorylated tau. Statistical significance was set at a *p*‐value  <  0.05.

## 3 RESULTS

### 3.1 Patient characteristics

**FIGURE 2:**
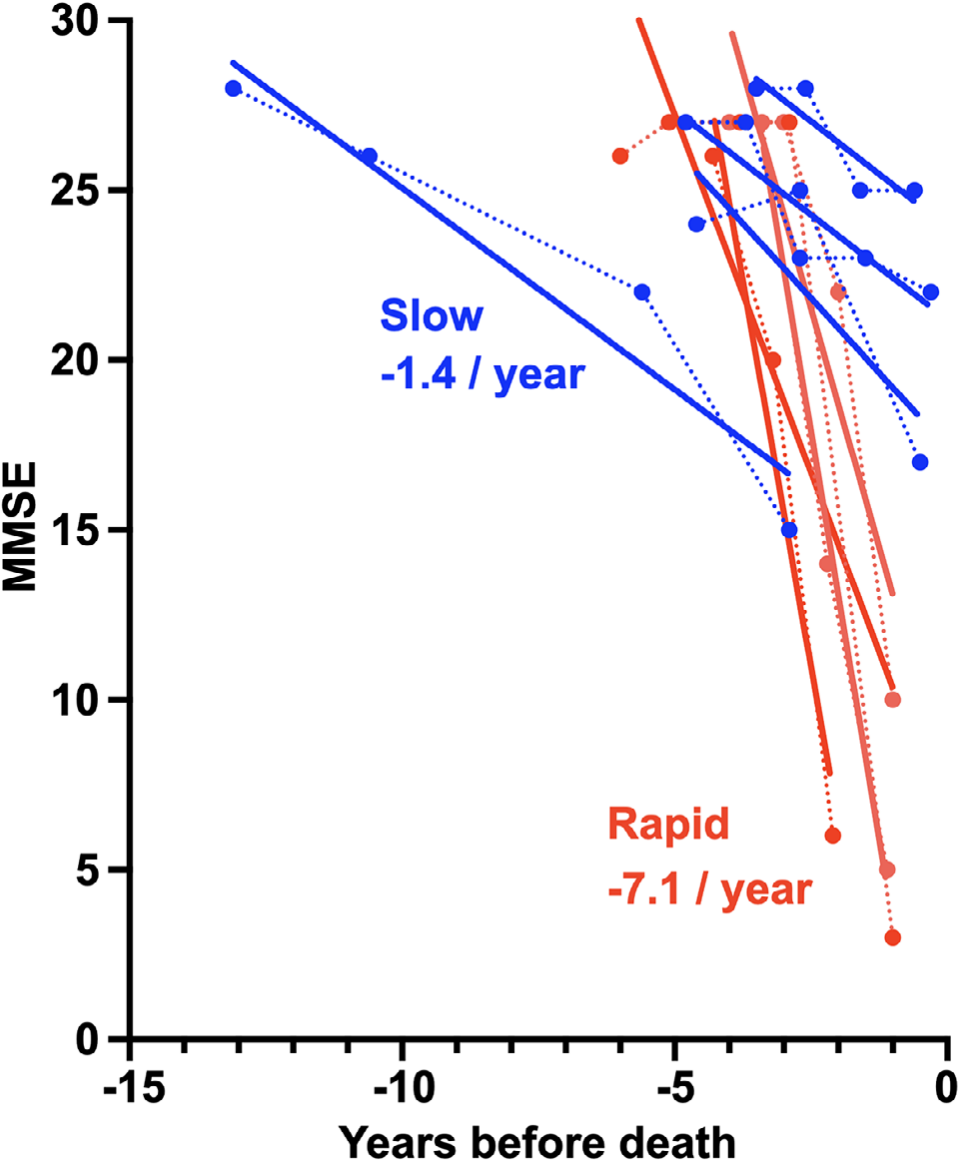
Longitudinal progression of cognitive decline. Individual MMSE scores are plotted for each patient, with dotted lines connecting sequential scores. The *x*‐axis represents years before death, with 0 indicating the time of death. Individual solid lines represent linear regression lines fitted to each patient's MMSE scores over time. Red and blue indicate rapid and slow decline groups, respectively. MMSE, Mini‐Mental Status Examination.

We identified 69 patients with DLB who underwent longitudinal evaluations at Mayo Clinic Jacksonville, came to autopsy at the Mayo Clinic brain bank between 2000 and 2021, and were neuropathologically confirmed as Lewy body disease. Based on the calculated annual rate of change of MMSE, we selected four patients from each end of the distribution to represent rapid and slow cognitive decline groups. As shown in Figure 2, the median annual MMSE change was a decline of 7.4 points in the rapid group and a decline of 1.2 points in the slow group.

Demographic, clinical, and neuropathologic characteristics of patients included in this study are summarized in Table 1. The rapid decline group consisted of four patients with diffuse Lewy body disease, while the slow decline group included three patients with diffuse Lewy body disease and one with transitional Lewy body disease. The mean interval between the last MMSE and death was 1.2 ± 0.9 years for the entire cohort and did not differ between rapid and slow decline groups (*p* = 0.75). Regarding anti‐dementia medications, 75% of patients in the rapid cognitive decline group were taking cholinesterase inhibitors, while in the slow cognitive decline group, 100% were on cholinesterase inhibitors, and 25% were taking N‐methyl‐D‐aspartate (NMDA) antagonists (full medication list is provided as Table S1). Rapid and slow decline groups also did not differ in age at death, disease duration, brain weight, Braak tangle stage, Thal amyloid phase, APOE e4 genotype, or comorbid pathologies, including TDP‐43 pathology and small vessel disease.

**TABLE 1 alz70374-tbl-0001:** Demographic, clinical, and pathological characteristics.

Features	Rapid decline (*n* = 4)	Slow decline (*n* = 4)	*p*‐Value
Females	1 (20%)	0 (0%)	0.999
MMSE annual decline, points	7.4 (5.2, 9.3)	1.2 (1.2, 1.4)	0.029
Age at death, yrs	73 (67, 79)	78 (74, 81)	0.686
Disease duration, yrs	6.8 (6.2, 8.1)	7.7 (7.3, 10.0)	0.400
Last MMSE to death interval, yrs	1.0 (1.0, 1.3)	0.5 (0.4, 1.2)	0.286
Cholinesterase inhibitors	3 (75%)	4 (100%)	0.999
NMDA receptor antagonists	0 (0%)	1 (25%)	0.999
Brain weight (g)	1170 (1110, 1250)	1290 ± 170	0.486
Diffuse LBD	4 (100%)	3 (75%)	0.999
Transitional LBD	0 (0%)	1 (25%)	0.999
Braak NFT stage	IV (III, V)	II (II, IV)	0.057
Thal amyloid phase	4 (3, 5)	2 (1, 4]	0.057
Small vessel disease	1 (20%)	1 (20%)	0.999
TDP‐43 pathology	0 (0%)	0 (0%)	–
*APOE ε4* carriers	3 (75%)	2 (50%)	0.999

*Note*: Data are presented as number of patients (%) or median (25th, 75th percentile).

Abbreviations: APOE, apolipoprotein E; LBD, Lewy body disease; MMSE, Mini‐Mental Status Examination; NFT, neurofibrillary tangle; TDP‐43, transactive response DNA binding protein of 43 kDa; yrs, years.

The degree of neuronal loss was generally minimal, remaining mild at most. No significant differences in neuronal loss were observed between the rapid cognitive decline and slow cognitive decline groups across any brain regions examined (Table S2).[Table alz70374-tbl-0001]

### 3.2 αSYN oligomers

**FIGURE 3 alz70374-fig-0003:**
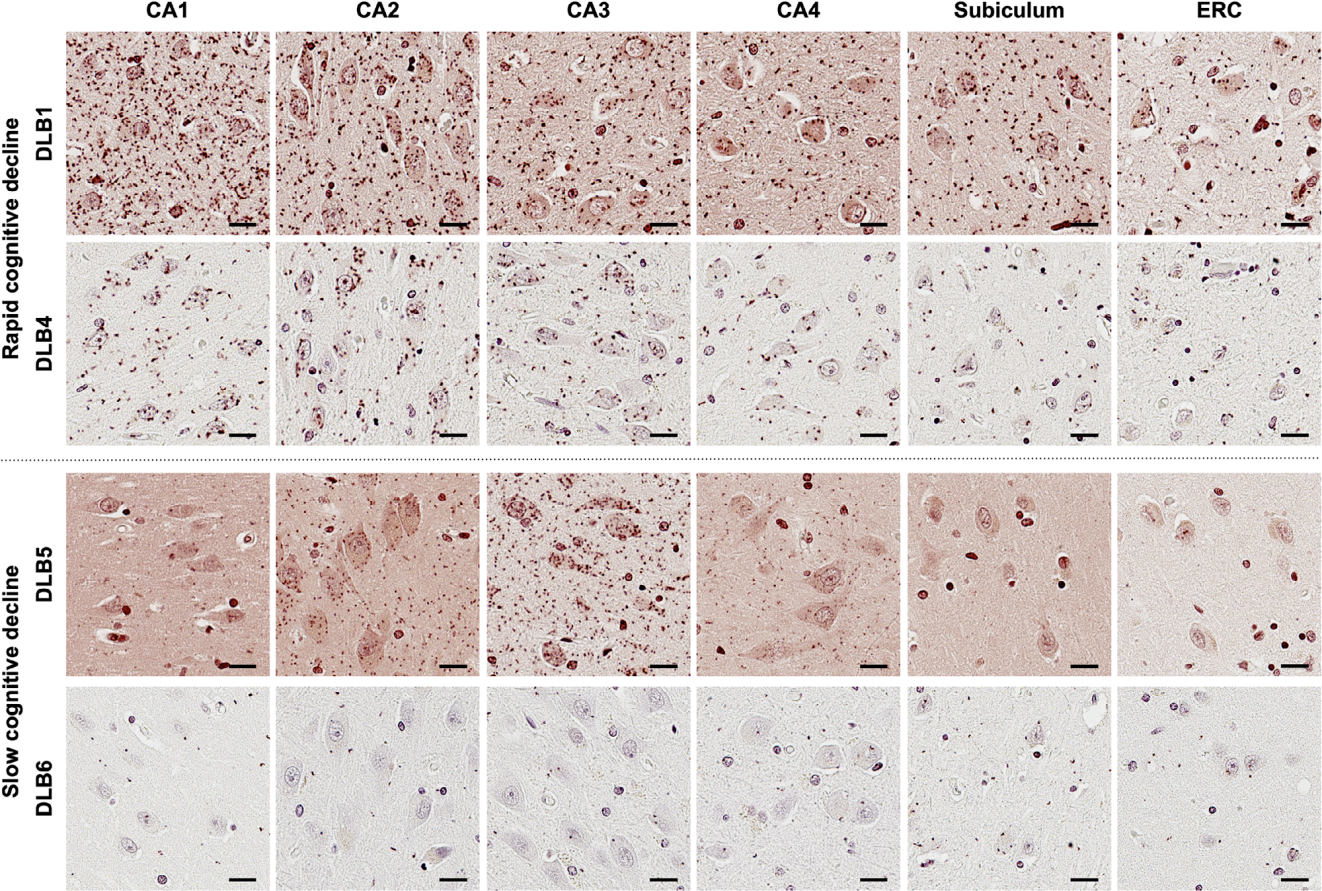
Representative images of αSYN‐PLA staining. The upper two rows show patients with rapid cognitive decline, while the lower two rows show patients with slow cognitive decline. Reddish‐brown dots indicate αSYN oligomers. Patients from the rapid decline group exhibit higher oligomer accumulation in neurons and neuropil of CA1 and CA2 subfields compared to the patients from the slow decline group. αSYN, α‐synuclein; ERC, entorhinal cortex; PLA, proximity ligation assay.

Figure 3 shows representative images of αSYN‐PLA staining in each brain region. The upper two patients demonstrated rapid cognitive decline, while the lower two patients had slow cognitive decline. Reddish‐brown dots indicate αSYN oligomers. Overall, patients with rapid decline had more abundant αSYN oligomers compared to those with slow decline. In CA1 and CA2 subfields of the hippocampus, patients with rapid cognitive decline had numerous clusters of αSYN oligomers not only in the neuropil, but also in the neuronal perikarya.

### 3.3 Neuropathologic burden

**FIGURE 4 alz70374-fig-0004:**
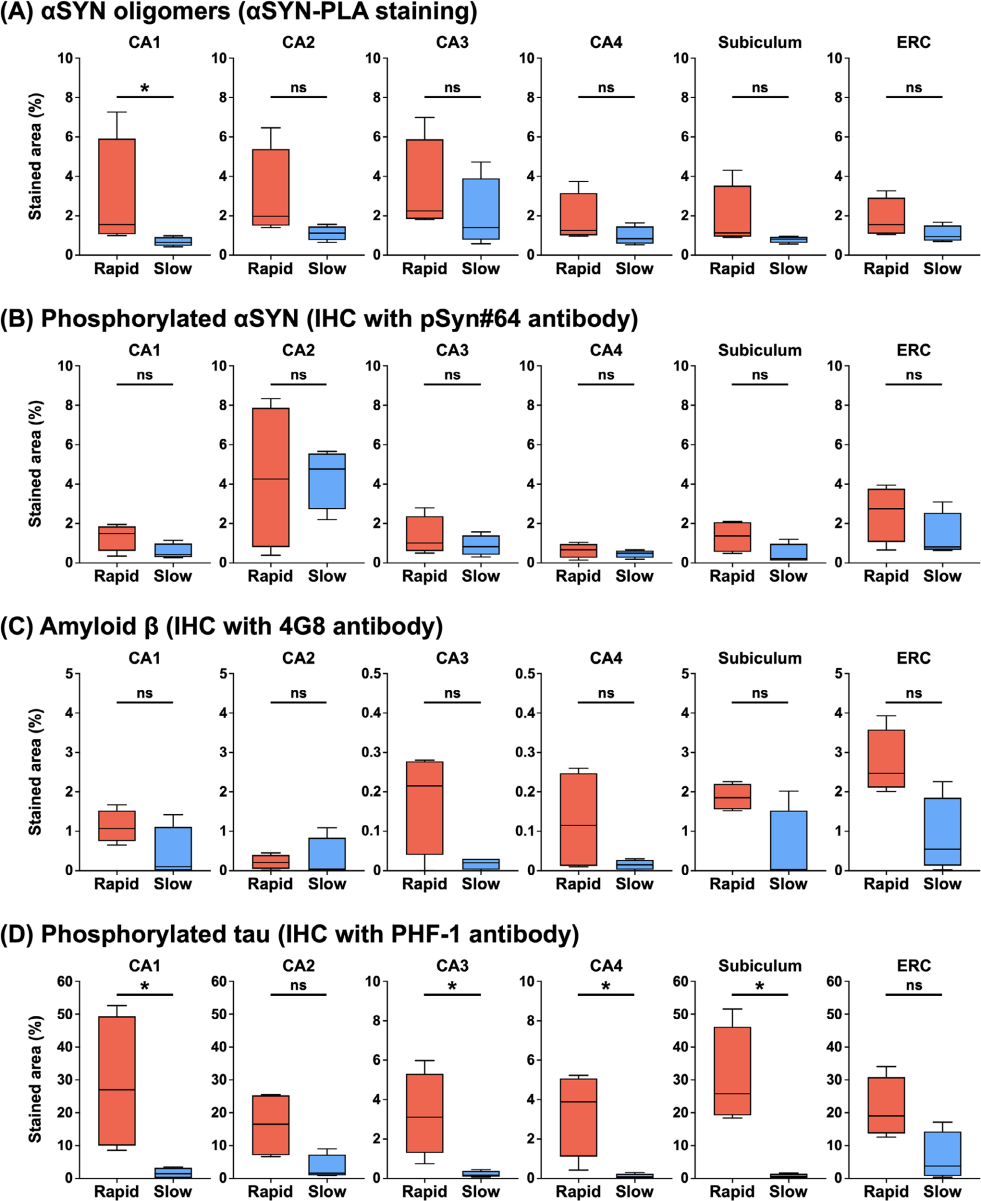
Comparison of the stained areas. Box plots compare the stained areas between rapid and slow cognitive decline groups across brain regions for (A) αSYN oligomers, (B) phosphorylated αSYN, (C) amyloid β, and (D) phosphorylated tau. The rapid decline group show significantly larger stained areas for αSYN oligomers in CA1, and for phosphorylated tau in CA1, CA3, CA4, and subiculum compared to the slow decline group. αSYN, α‐synuclein; ERC, entorhinal cortex; IHC, immunohistochemistry.PLA, proximity ligation assay.

Figure 4 summarizes the stained area for each stain. The immunostaining areas of phosphorylated tau were greater in the rapid decline group compared to the slow decline group in CA1, CA3, CA4, and subiculum (*p* = 0.029). The quantitative burden of amyloid‐β did not differ in hippocampal subfields or the subiculum between rapid and slow decline groups. The rapid cognitive decline group had greater αSYN oligomer burden in the CA1 sector of the hippocampus compared to the slow cognitive decline group (median %area 1.56 vs. 0.65; *p* = 0.029). A trend toward higher αSYN oligomer burden in the CA2 region was observed in the rapid cognitive decline group compared to the slow cognitive decline group, although this difference did not reach statistical significance (median %area 1.97 vs. 1.11; *p* = 0.057). In contrast, the neuropathologic burden of mature phosphorylated αSYN did not differ in any brain regions.

To investigate the relationship between αSYN oligomers and phosphorylated tau, we examined the correlation between their respective stained areas (FigureS1). Significant positive correlations were observed between these proteins across many brain regions. Notably, high positive correlations (*r* = 0.95, *p* = 0.001) were found between αSYN oligomers in CA1 and phosphorylated tau in CA1, CA3, and CA4.

## 4 DISCUSSION

The present study demonstrated that the accumulation of αSYN oligomers and phosphorylated tau was greater in the hippocampus in DLB with rapid cognitive decline compared to those with slow cognitive decline. While previous studies have investigated how various neuropathologic changes, including AD pathology, contribute to cognitive decline, the pathogenesis of cognitive impairment remains complex and not fully explained.^22^ Although αSYN oligomers, which are considered early aggregates of αSYN, were shown to be highly toxic,^7^, ^8^ they can be difficult to visualize, and their clinicopathologic correlations have not been thoroughly elucidated. Our previous research demonstrated an association between hippocampal αSYN oligomers and cognitive decline in Parkinson's disease.^11^ Similarly, in multiple system atrophy, a neuroglial α‐synucleinopathy, hippocampal αSYN oligomers correlated with cognitive impairment.^12^ The current results provide more evidence of the toxicity of αSYN oligomers, by examining whether they are associated with a steeper cognitive trajectory. We found that patients with rapid cognitive decline had significantly higher levels of αSYN oligomers in CA1 and demonstrated a trend toward increased αSYN oligomers in CA2 and subiculum compared to patients with slow cognitive decline. In contrast, the burden of phosphorylated αSYN, which is considered to represent mature Lewy‐related pathology, did not differ between the two groups in the regions studied. Previous studies using cell cultures and animal models have shown that αSYN oligomers exhibit stronger toxicity than Lewy‐related pathology, which consists of phosphorylated αSYN aggregates.^7^, ^8^ Various mechanisms of αSYN oligomer toxicity have been proposed, including synaptic dysfunction, membrane disruption, mitochondrial impairment, endoplasmic reticulum stress, and oxidative stress.^23^ Our finding of increased αSYN oligomers in the hippocampus of patients with rapid cognitive decline provides evidence from human autopsy brains that αSYN oligomers may influence the clinical progression of the disease. The accumulation of αSYN oligomers in the hippocampus observed in our study may contribute to accelerated cognitive decline through one or more of these toxic mechanisms. Further research is warranted to determine which of these mechanisms predominantly contributes to cognitive deterioration.[Fig alz70374-fig-0003]

Our findings that αSYN oligomers, rather than Lewy‐related pathology, are associated with the rate of cognitive decline may seem counterintuitive given that αSYN oligomers eventually transform into Lewy‐related pathology during the aggregation process. However, this observation is consistent with growing evidence suggesting that oligomers represent the most neurotoxic form of αSYN, as previously discussed. Furthermore, it has been hypothesized that the formation of Lewy‐related pathology may represent a cellular protective response that sequesters more toxic oligomeric species.^24^, ^25^ This could explain why the burden of phosphorylated αSYN (Lewy‐related pathology) did not differ between the two groups despite significant differences in αSYN oligomer burden. A temporal shift in αSYN aggregation^11^ may also be involved. Oligomeric species represent the early stages of aggregation and may better reflect active pathologic processes causing cognitive decline, whereas Lewy‐related pathology accumulates over longer periods and therefore may not correlate as strongly with the rate of clinical progression. Thus, our results support the hypothesis that αSYN oligomers, rather than Lewy‐related pathology, are the primary drivers of cognitive decline in DLB.

In DLB, a more rapid cognitive trajectory occurs in those with greater Lewy‐related and AD co‐pathology as assessed by Braak tangle stages and neuritic plaques.^26^, ^27^ We examined whether the severity of AD‐related pathology may also be related to cognitive decline. The extent of amyloid‐β did not differ between rapid and slow decliners, suggesting that amyloid‐β has a limited impact on the rate of cognitive decline in DLB. Nonetheless, phosphorylated tau was more abundant in CA1, CA3, CA4, and subiculum regions in the group showing a steeper cognitive decline compared to those with a more gradual decline. These findings add to the evidence that αSYN oligomers and phosphorylated tau pathology contribute to cognitive trajectory in DLB. The strong positive correlation observed between αSYN oligomers and phosphorylated tau might suggest a potential synergistic interaction between these pathologies. Due to the limited sample size in this study, multivariable analysis was not feasible, and we could not conclude which pathology, αSYN oligomers or phosphorylated tau, contributed most significantly to cognitive decline. Future studies are needed with a larger cohort, such as one with matched tau burden, to determine whether αSYN oligomers and phosphorylated tau pathology have an independent or synergistic effect on cognitive progression.[Fig alz70374-fig-0004]

Mesial temporal tau deposition in normal aging is a predictor of memory impairment,^28^ and tau deposition in the CA1 sector of the hippocampus is affected early and severely in AD,^29^ and is associated with memory loss.^30^ The CA1 sector of the hippocampus shows better preservation in DLB,^31^ but when tau deposition does occur in this hippocampal subfield, there is evidence of concurrent Lewy‐related pathology, each of which contributes independently to memory impairment.^32^, ^33^ Our data also show that tau and Lewy‐related pathology can accumulate in the CA1 region in DLB, but when examined separately, only tau was associated with a steep dementia trajectory in our small sample. This discrepancy may be attributed to two key methodologic differences. First, Adamowicz and colleagues^27^ examined pathology as predictors of baseline memory performance, whereas our study focused on the contribution of pathology to the trajectory of cognitive decline. Second, their study had a longer interval between cognitive assessment and death (mean of 6 years), suggesting that the neuropathologic findings at autopsy might not accurately reflect the brain pathology present at the time of cognitive testing. Our study, with a shorter interval between the final MMSE and death (approximately 1 year), may provide a more direct assessment of the pathological correlates with cognitive decline.

A major limitation of the present study is the small sample size which affects the power to examine multivariable contributions of continuous variables. Our results should be interpreted as preliminary evidence that requires validation in larger cohorts. Future studies with expanded sample sizes will be essential to confirm and extend our findings. While this sample size is limited, we applied rigorous inclusion criteria to ensure the quality and comparability of our cases. The statistically significant differences observed despite the small sample size suggest a robust biological effect of αSYN oligomers on cognitive decline in DLB. Additionally, our study design focused on comparing the extreme ends of the cognitive decline spectrum, potentially missing insights from patients with intermediate rates of decline. Nevertheless, the cohort consisted of well‐characterized patients who underwent prospective longitudinal neuropsychological assessments and had neuropathologically confirmed diagnoses. Importantly, the average interval between the last neuropsychological assessment and death was approximately 1 year, suggesting that the observed neuropathologic changes are reflective of the clinical course.

In conclusion, this study demonstrates that hippocampal αSYN oligomer accumulation in the CA1 region, along with phosphorylated tau pathology, may contribute to rapid cognitive decline in DLB patients. This provides a new insight into the relationship between αSYN oligomers and cognitive decline using human postmortem brain tissue. Further investigation is needed to determine whether targeting αSYN oligomers could be a potential therapeutic strategy to modify the trajectory of cognitive decline in DLB.

## CONFLICT OF INTEREST STATEMENT

The authors report no conflict of interest to disclose. Author disclosures are available in the Supporting Information.

## CONSENT STATEMENT

Brain autopsies were conducted with the consent of the legal next‐of‐kin or an individual with legal authority to grant permission for autopsy. De‐identified studies using autopsy samples are considered exempt from human subject research by the Mayo Clinic Institutional Review Board.

## Supporting information



Supporting Information

Supporting Information
