# Supplementary material for: Impact of hippocampal α‐synuclein oligomers on cognitive trajectory in patients with dementia with Lewy bodies
Source: Alzheimers Dement. 2025 Aug 4;21(8):e70374. doi: 10.1002/alz.70374 (PMC12319237; doi:10.1002/alz.70374)
Supplement: Supplementary file 2 — Supporting Information [file ALZ-21-e70374-s002.pdf]

Supplementary material

Impact of Hippocampal α-Synuclein Oligomers on Cognitive Trajectory in Patients with Dementia with Lewy Bodies

Hiroaki Sekiya, M.D., Ph.D.[1], Lukas Franke, M.D.[1], Daisuke Ono, M.D., Ph.D. [1], Michael DeTure, Ph.D.[1], Owen A. Ross, Ph.D.[1], Melissa E. Murray, Ph.D.[1,2], Pamela J. McLean, Ph.D.[1], Tanis J. Ferman, Ph.D.[3], Dennis W. Dickson, M.D.[1,2]

[1] Department of Neuroscience, Mayo Clinic, 4500 San Pablo Rd, Jacksonville, Florida 32224, USA

[2] Department of Laboratory Medicine and Pathology, Mayo Clinic, 4500 San Pablo Rd, Jacksonville, Florida 32224, USA

[3] Department of Psychiatry & Psychology, Mayo Clinic, 4500 San Pablo Rd, Jacksonville, Florida 32224, USA

*Corresponding author: Hiroaki Sekiya, MD, PhD

Supplementary Figure 1

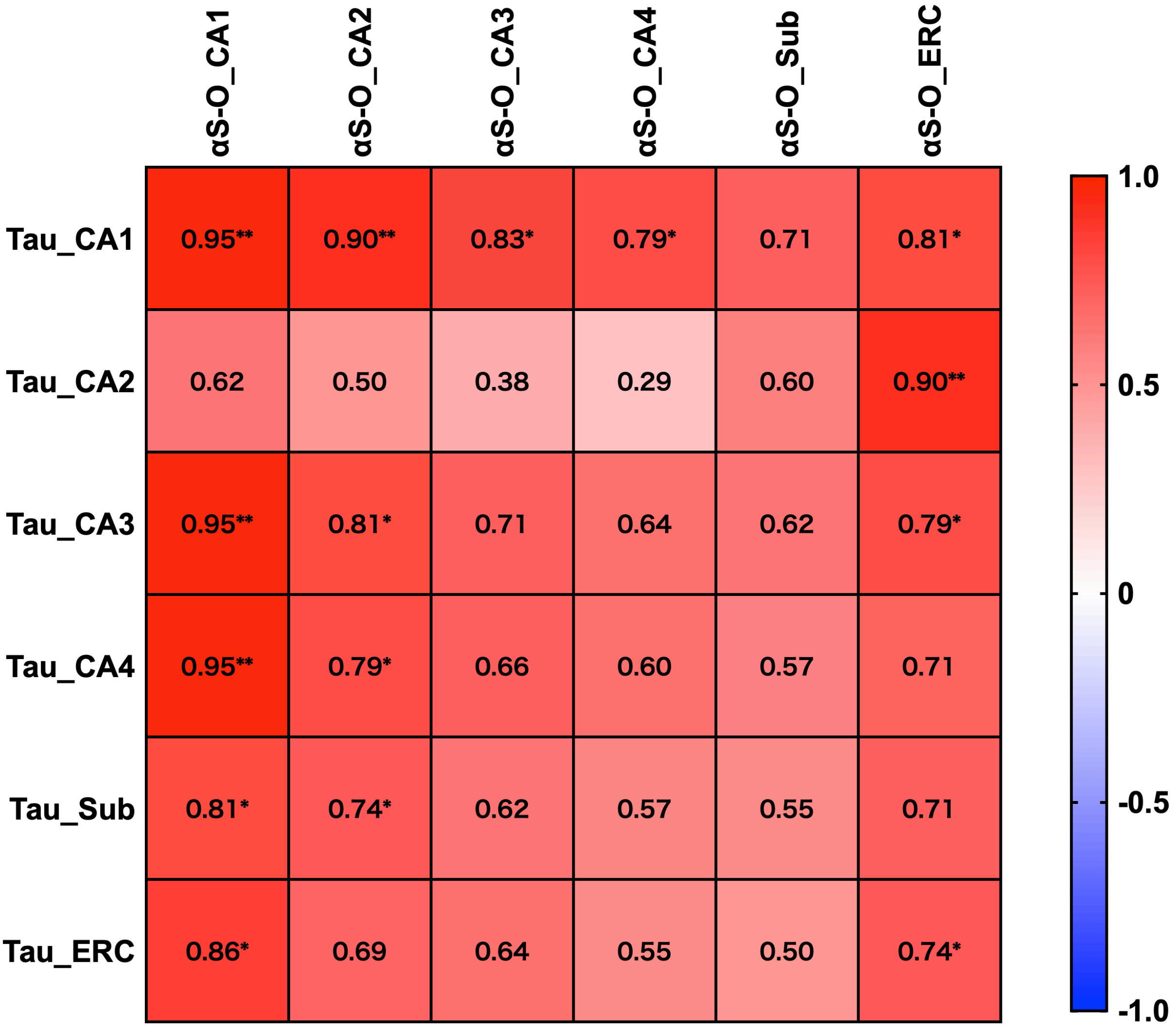

Association between phosphorylated tau and α-synuclein oligomer burden.

Spearman's rank correlation coefficient was calculated to examine the association between the stained areas of phosphorylated tau and α-synuclein oligomers. Positive correlations were observed between these pathological burdens in multiple regions.

αS-O, α-synuclein oligomer; *ERC*, entorhinal cortex; *Sub*, subiculum; *tau*, phosphorylated tau; * p<0.05; ** p<0.01

Supplementary Table 1 Medication list

ID	Group	Sex	AAD	Medications
DLB1	Rapid	F	68	Donepezil, carbidopa/levodopa, mirtazapine, levothyroxine, cyanocobalamin, omeprazole, propranolol
DLB2	Rapid	M	77	Carbidopa/levodopa, quetiapine, bupropion, buspirone, amlodipine
DLB3	Rapid	M	65	Rivastigmine, carbidopa/levodopa, quetiapine, clonazepam, escitalopram, tamsulosin, midodrine
DLB4	Rapid	M	85	Donepezil, quetiapine, mirtazapine, sertraline, tamsulosin, finasteride
DLB5	Slow	M	80	Donepezil, carbidopa/levodopa, venlafaxine, finasteride, atorvastatin, melatonin
DLB6	Slow	M	75	Donepezil, memantine, carbidopa/levodopa, rasagiline
DLB7	Slow	M	72	Donepezil, carbidopa/levodopa, venlafaxine, metoprolol, rosuvastatin, warfarin
DLB8	Slow	M	82	Donepezil, carbidopa/levodopa, zolpidem, losartan, hydrochlorothiazide, glimepiride, tolterodine, calcium carbonate, polyethylene glycol, terbinafine, ketoconazole

AAD, age at death; *Rapid*, rapid cognitive decline; *Slow*, slow cognitive decline

Supplementary Table 2 Neuronal loss in each brain region

Brain regions	Rapid decline (n = 4)	Slow decline (n = 4)	p-value
CA1	0.5 (0, 1)	0 (0, 0)	0.429
CA2	0 (0, 0)	0 (0, 0)	0.999
CA3	0 (0, 0)	0 (0, 0)	0.999
CA4	0 (0, 0)	0 (0, 0)	0.999
Subiculum	0.5 (0, 1)	0 (0, 0)	0.429
Entorhinal cortex	0.5 (0, 1)	0 (0, 0.75)	0.999

Data are presented as median (25th, 75th percentile).
